# Heteroaryl-Fused Triazapentalenes: Synthesis and Aggregation-Induced Emission

**DOI:** 10.3390/molecules30010156

**Published:** 2025-01-03

**Authors:** Yingchun Wang, Thanh Chung Pham, Jianjun Huang, Junfeng Wu, Wim Dehaen

**Affiliations:** 1 Henan Key Laboratory of Water Pollution Control and Rehabilitation Technology, Henan University of Urban Construction, Pingdingshan 467036, China; wangyc0628@163.com; 2 Sustainable Chemistry for Metals and Molecules, Department of Chemistry, KU Leuven, Celestijnenlaan 200F, 3001 Leuven, Belgiumjianjun.huang@kuleuven.be (J.H.)

**Keywords:** pyridine-fused triazapentalene, modified Suzuki cross-coupling, CH arylation, fluorescent properties, solid-state, aggregated state

## Abstract

A pyridine-fused triazapentalene shows weak fluorescence in solution and is readily accessible via nitrene-mediated cyclization. In this study, a modified Cadogan reaction was used to synthesize **HetATAP 1**. Palladium-catalyzed reactions have been used as post-functionalization methods. Interestingly, modified Suzuki cross-couplings with various boronic acids resulted in poor to moderate yields of **HetATAPs 2**–**5** which were arylated at the azole moiety. Direct CH arylation of **HetATAP 1** gave the products with the same regiochemistry in satisfactory yields. The structures of **HetATAPs 2**–**5** were confirmed using NMR analysis. In addition, the photophysical properties of **HetATAPs 1**–**5** were studied under various conditions. Particularly, the emission of **HetATAPs 2**–**5** is enhanced in the solid and aggregate state.

## 1. Introduction

**Scheme 1:**
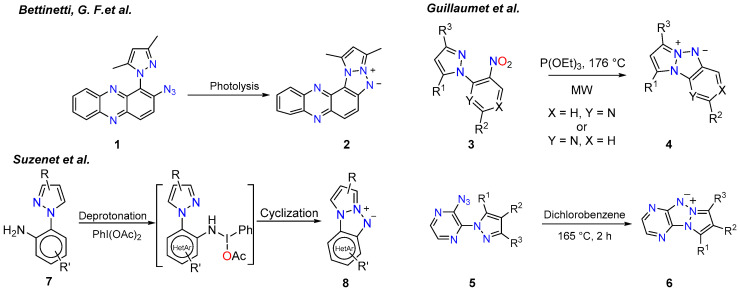
Synthetic pathways toward heteroaryl-fused triazapentalenes (**HetATAPs**) [2,3,6,7,10,11] and the numbering of the triazapentalene (TAP) core.

Heteroaryl-fused triazapentalenes (**HetATAPs**), more specifically *N*-heteroaryl triazapentalenes (***N*-HetATAPs**), show fluorescent properties that are of interest for potential applications [1,2,3,4,5,6,7]. Currently, only two general synthetic pathways toward HetATAPs have been reported [2,3,8,9,10,11], as shown in Scheme 1. The first published procedure mostly focuses on the deoxygenation of 1-(o-nitro(hetero)aryl)pyrazoles and the thermolysis or photolysis of 1-(o-azido(hetero)aryl)pyrazoles [10,11]. In particular, **HetATAP** has been studied by the Bettinetti group, forming in unreactive media starting from 2-azido-1-(3,5-dimethylpyrazol-1-yl)phenazine **1**. The triazapentalene **2** was produced by intramolecular trapping of the formed nitrene. On the other hand, the synthetic methodology of Guillaumet et al. involves a deoxygenation reaction using nitro(pyrazolyl)pyridines **3**, which leads to pyridine-fused triazapentalenes **4 [3]**. Although the deoxygenation reaction was only slightly influenced by the substituents at the pyrazole core, the modification at the nitropyridine part had a considerable impact on the reaction result. Later in 2009, the same group reported a library of pyrazine-fused triazapentalenes **6** by the thermolysis of 2-azido-3-(pyrazol-1-yl)pyrazines **5**, starting from nitropyrazoles and 2-azido-3-chloropyrazine [2]. The second reported pathway toward compound **8** begins with coupling reactions or nucleophilic aromatic substitutions with halogenated heterocyclic amines and pyrazole derivatives, followed by oxidative cyclization (Scheme 1, Suzenet et al.) [6,7]. Involving a radical mechanism under basic conditions in the presence of hypervalent iodine, the direct formation of an intramolecular N–N bond takes place to form the desired **HetATAP** derivatives. Unfortunately, in function of the substitutions on the fused ring and the position of the amine, the reaction yields were variable.

Applying the oxidative intramolecular N–N bond formation, Suzenet et al. studied the photophysical properties of HetATAP derivatives [6]. However, most exhibit modest to poor fluorescence quantum yields in solution. The variable quantum yields can be attributed to either the charge of the nitrogen atoms in the heteroaryl moiety or substituents in the azole moiety. Introducing two nitrogen atoms in the fused ring and/or an electron-withdrawing group on the azole ring in the case of R^3^ position can improve the quantum yields of the resulting dyes in solution [6,7].

In 2001, Tang et al. reported that the aggregation of silole molecules induced light emission, which was opposite to the well-known aggregation-caused quenching (ACQ) [12]. Aggregation-induced emission enhancement (AIEE) has sparked considerable interest because of its broad potential applications in physics [13], chemistry [14], biology [15,16,17,18], and material science [19,20,21,22]. However, as the formation of delocalized excitons or excimers may lead to enhanced non-radiative inactivation of the excited state, most organic molecules show fluorescence ACQ in their aggregated or solid state. The reported organic fluorescence dyes exhibit AIEE characteristics, such as siloles [12,23,24,25], tetraphenylethylene (TPE) [26,27], 1-cyano-trans1,2-bis(4′-methylbiphenyl)ethylene (CN-MBE) [28], 1,4-di[(E)-2-phenyl-1-propenyl]benzene (PPB) [29,30], and salicylaldehyde azine derivatives [31,32].

To date, the photophysical properties of **HetATAP** derivatives have only been reported in solution. The successful applications of **HetATAPs** are limited by their use as fluorescent probes in living cells and fluorescent dyes inserted into LDH host structures and polymers [1,5,7,33]. Because of the growing interest in **HetATAP** fluorophores, the initial goal of the present work was to expand the variety of substitution patterns on the heteroaryl moiety of triazapentalenes via post-functionalization strategies and study the photophysical properties of **HetATAP** in solids. Furthermore, a few reports mention that the benzotriazole moiety in the molecular structure could be used as an acceptor in donor–acceptor–donor (D–A–D) conjugated systems [34] and a triazole-based probe has also been used for optical imaging of cellular targets with high Stokes shifts [35]. For this purpose, the readily available Cadogan reaction was chosen as the synthetic pathway to obtain **HetATAP 1**, and the feasibility of the palladium-catalyzed cross-coupling reactions was demonstrated. Herein, we describe the synthesis of **HetATAP** derivatives and their fluorescence emission in solid-state and aggregate conditions. To the best of our knowledge, the photophysical properties of solid/aggregate-state **HetATAPs** have not been reported until now [1].

## 2. Results and Discussion

### 2.1. Synthesis

**Scheme 2:**
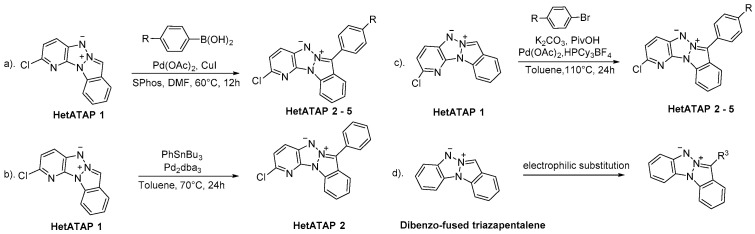
The synthetic pathways towards **HetATAPs 2**–**5**. (**a**). Palladium-catalyzed reaction 1, (**b**). Palladium-catalyzed reaction 2, (**c**) Palladium-catalyzed reaction 3, (**d**). Electrophilic substitution of dibenzo-fused triazapentalene.

To start our investigation, the desired **HetATAP 1** was obtained using a modified Cadogan reaction (see SI Scheme S1) [3]. In an attempt to arylate heteroaryl-fused triazapentalene, procedures previously reported for the arylation of 1,3a,6a-triazapentalene with palladium catalysis were applied to **HetATAP 1** [36]. The reported procedure with a modified Suzuki cross-coupling reaction (palladium-catalyzed reaction 1) was first carried out taking advantage of the presence of a chlorine atom on the pyridine moiety and was performed with one equivalent of phenylboronic acid in the presence of CuI in DMF at 60 °C for 12 h. Surprisingly, a CH arylation occurred instead of the excepted coupling reaction (Scheme 2a). The unexpected compound **HetATAP 2** was formed and its structure was confirmed via NMR analysis. In addition, we have sequentially attempted a modified Stille coupling reaction (palladium-catalyzed reaction 2) with PhSnBu_3_ and direct palladium-catalyzed CH arylation with bromobenzene (palladium-catalyzed reaction 3) using toluene as solvent, which provided the same product (Scheme 2b,c). Similar reaction yields were obtained for **HetATAP 2** from the palladium-catalyzed reaction 1 and 3, but only 13% from the palladium-catalyzed reaction 2. Hence, to extend the substituents, the palladium-catalyzed reactions 1 and 3 were carried out with different boronic acids and bromoarenes, respectively (Table 1). The reaction yields of the palladium-catalyzed reaction 3 were quite satisfying, and consequently higher than those of palladium-catalyzed reaction 1. In 2008, Gorelsky et al. reported the mechanism of the direct CH bond cleavage in the presence of Pd(OAc)_2_ and potassium carbonate for the (electron-rich) heteroarenes [37]. As mentioned above, the direct palladium-catalyzed CH arylation conditions were adopted for the arylation of 1,3a,6a-triazapentalene, where arylation may occur via a concerted metalation-deprotonation in the presence of carbonate. For this process, the same reaction pathway is considered to have happened. Furthermore, the substitution position was the same as the one of dibenzo-fused triazapentalenes (Scheme 2d). We assume that adding a nitrogen atom in the fused ring would not be enough to change the charge characteristics at each location of the triazapentalene core.

### 2.2. Photophysical Measurements

#### 2.2.1. Fluorescence in the Solid-State

**Figure 1:**
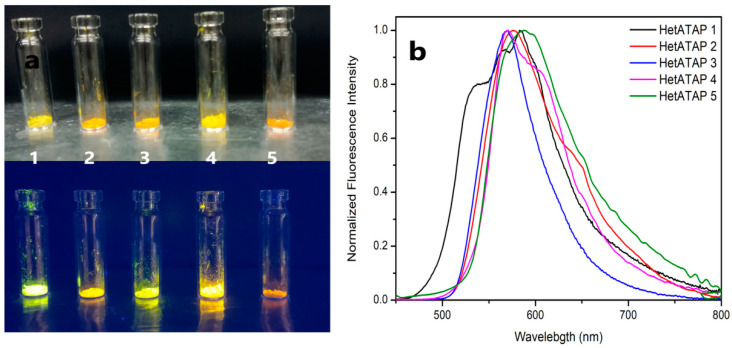
The color of compounds observed with the naked eye and by irradiation with a UV lamp (**a**) (365 nm) and normalized fluorescence emission spectra of **HetATAPs 1**–**5** in the solid-state (**b**).

The absorption and emission spectra were taken for **HetATAP 1** and its postmodified products **HeTATAPs 2**–**5** in solid-state at room temperature. The colors of these compounds under the naked eye and a UV lamp are collected in Figure 1a, and the emission spectra for **HetATAPs 1**–**5** are shown in Figure 1b. The photophysical parameters are listed in Table 2. The color of the compounds shows slight differences. Observing the brightness of all compounds under UV light, **HetATAPs 2** and **5** are less intense compared to **HetATAP 1**, while **HetATAPs 3** and **4** are slightly more intense, suggesting different fluorescence quantum yields (Φ_F_, 5–20%) which were obtained by the directed measurement with integrating spheres (Table 2). Compared to the precursor **HetATAP 1**, the absorption spectra of **HetATAPs 2**–**4** are redshifted and the emission spectra are slightly blue-shifted. Only **HetATAP 5** (R=N(CH_3_)_2_) exhibited an absorption band at 458 nm and an emission band at 588 nm, which was assigned to the electron-rich character of the TAP core. The presence of additional functional groups that could disturb the molecular planarity of **HetATAP 1** might be responsible. In **HetATAPs 2**–**5**, the Stokes shift is smaller than that of **HetATAP 1**, suggesting more coplanarity. 

#### 2.2.2. Spectral Properties in Solution

**Figure 2:**
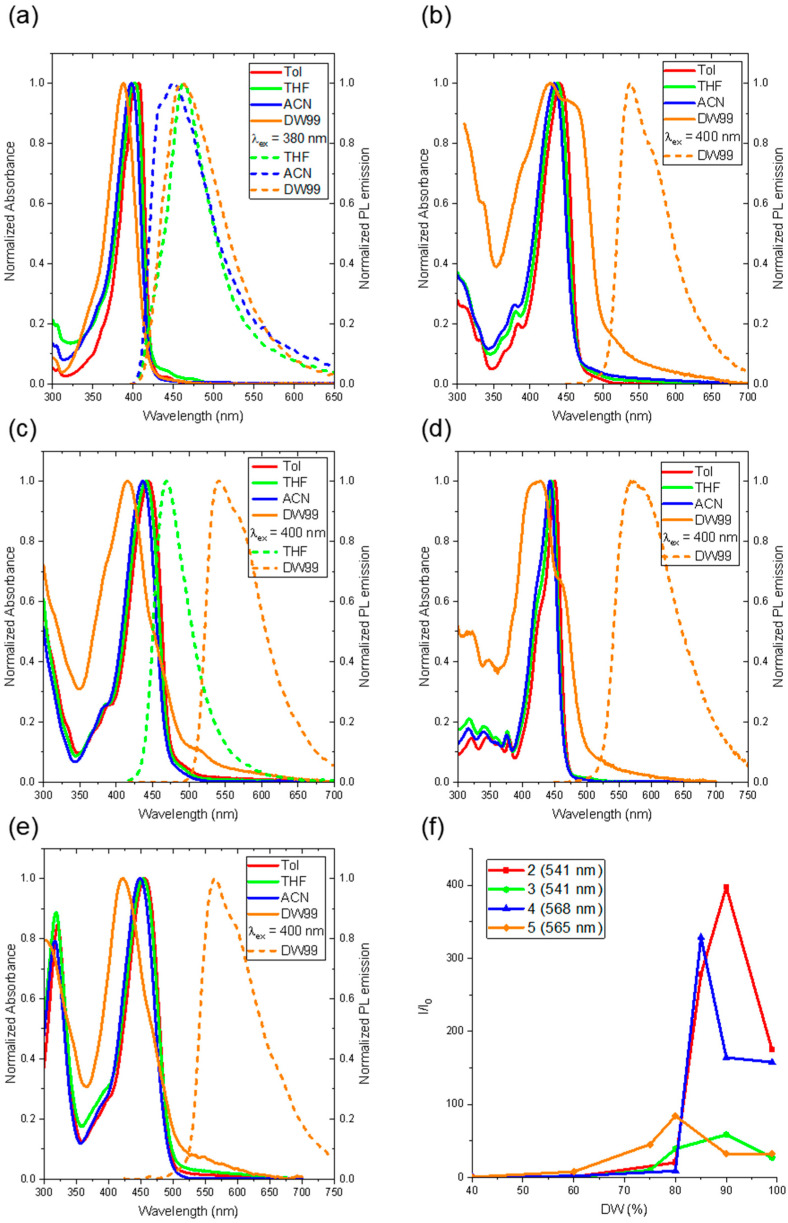
(**a**–**e**) Normalized UV–Vis absorption and fluorescence emission spectra of **HetATAPs 1**–**5** in toluene (Tol), tetrahydrofuran (THF), acetonitrile (ACN), and distilled water (1%THF) (DW99) (20 µM); (**f**) fluorescence emission intensity ration (I/I_0_) of **HetATAPs 2**–**5** (10 µM) in THF/DW (0–99%) solvent mixture.

The UV–Vis absorption and fluorescence emission spectra of **HetATAPs 1–5** in toluene (Tol), tetrahydrofuran (THF), acetonitrile (ACN), and distilled water (1%THF) (DW99), with a concentration of 20 µM, are shown in Figure 2. The respective photophysical properties data of **HetATAPs 1**–**5** for the solvents used in the study are presented in Table 3. All of the **HetATAP** derivatives show absorption spectra in a similar wavelength region that changes little with the variation in solvent (Table 3). Their absorption peaks (λ_abs_) appear at 388–456 nm, which were assigned to the π-π* electronic transitions of these molecules. With increasing solvent polarity (from Tol up to DW99), a general blue shift is observed, but the extent of the spectral shift is relatively small (12–33 nm).

All the **HetATAP** derivatives studied in this work showed negligible luminescence emission in organic solvents (e.g., Tol, THF, and ACN). The **HetATAP 1** exhibited similar emission bands (463 nm, 450 nm, 461 nm) in THF, can, and DW99, respectively (Table 3). Meanwhile, this compound displayed a very low fluorescent quantum yield (Ф_F_ ≤ 0.5%) and small Stokes shifts (5–71 nm) in various solvents. The luminescence behaviors of the **HetATAPs 2**–**5** solutions vary with the solvent interestingly. Only **HetATAP 3** exhibited a weak emission band at 469 nm (Ф_F_ = 0.1%) in THF, which was assigned as locally-excited (LE) emission of the TAP core. The fluorescent signals of all **HetATAP** derivatives in this study were detected in DW99. **HetATAP 2** and **HetATAP 3** gave the same emission peak ((λ_ems_ = 541 nm) and similar emission spectra (Figure 2b,c). Compared with **HetATAP 3** ((λ_ems_ = 541 nm, Ф_F_ = 2.3%), **HetATAP 2** displayed a relatively high fluorescent quantum yield (Ф_F_ = 8%) and smaller Stokes shift (112 nm) (Table 3). **HetATAP 4** and **HetATAP 5** show absorption peaks at 428 and 423 nm, respectively, suggesting that the former is more conjugated than the latter. Figure 2d,e show the emission spectra of **HetATAP 4** and **HetATAP 5**, respectively, and these both present green emissions. When these compounds were excited by 400 nm light, the maximum emission peaks of the compounds appeared at 568 nm and 565 nm, respectively. Further, the fluorescent quantum yield (Ф_F_) of **HetATAP 4** and **HetATAP 5** in DW99 solution was shown to be 13.1% and 2.6%. **HetATAP 4** (R = COOEt) exhibits the highest fluorescent quantum yield in DW99 (Table 3), suggesting that the electron-rich character of the TAP core and the electron-withdrawing group (EWG) with help the electron delocalization, favoring the resonance structure [38]. Furthermore, by comparing the fluorescence quantum yields of these compounds in solution Ф_F(solution)_ and solid-state Ф_F(solid)_, especially for those compounds with Ф_F(solid)_/Ф_F(solution)_ > 1, including **HetATAP 3**–**5**, we estimated that they might show effective AIE behavior. 

### 2.3. Fluorescence in the Aggregated State

**Figure 3:**
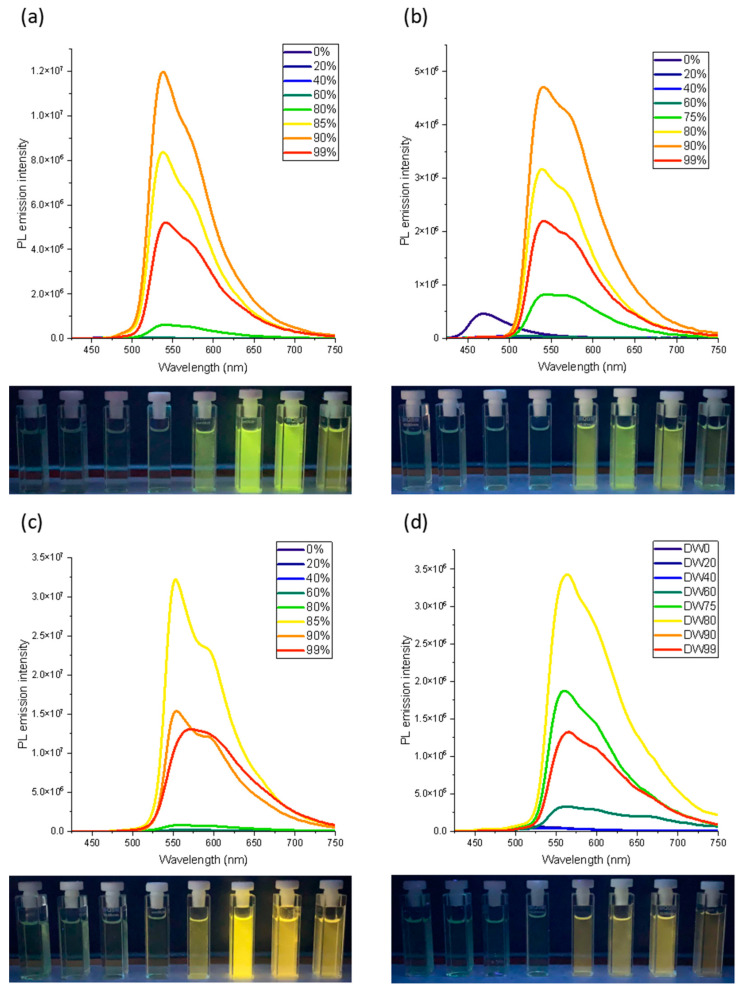
(**a**–**d**) Fluorescence emission spectra and image (under 365 nm light) of **HetATAPs 2**–**5** (10 µM) in THF/DW (0–99%) solvent mixture.

To verify the AIEE properties of **HetATAPs 2**–**5**, fluorescence emission spectra and images (under 365 nm light) were recorded in THF/water mixtures (Figure 2f and Figure 3). As shown in Figure 2f and Figure 3, weak fluorescent signals are seen at *f*_w_ ≤ 60% because the **HetATAPs 2**–**5** molecules are generally dissolved in these mixtures. The fluorescent intensity starts to rise for *f*_w_ > 60%, where the solvating power of the mixture is lowered to such an extent that the luminogen molecules begin to aggregate. As *f*_w_ reached 80% for **HetATAPs 2** and **4**, the fluorescent spectra showed a sharp increase compared to **HetATAPs 2** and **4** (Figure 2f). However, when *f*_w_ increased up to 80% (**HetATAP 5**), 85% (**HetATAP 4**), or 90% (**HetATAPs 2** and **3**), the emission exhibited a decreasing trend owing to more aggregates precipitated might form during this process. To summarize, **HetATAPs 1**–**4** demonstrates either no or minimal LE fluorescence emission (e.g., **HetATAP 3** in THF) under non-aggregated conditions. However, fluorescence emission is significantly enhanced in the presence of aggregated states, which can be assigned to the TICT effect.

## 3. Materials and Methods

### 3.1. Chemical and Materials

All chemicals were purchased from Acros Organics (Geel, Belgium), Merck (Boston, MA, USA), J&K Scientific (Beijing, China), Fluorochem (Derbyshire, UK), and TCI Europe (Antwerp, Belgium) and used as received. Solvents were dried prior to use. For column chromatography, 70–230 mesh silica gel 60 (Acros) was used as the stationary phase.

### 3.2. Instrumentation

^1^H and ^13^C NMR spectra were recorded on a Bruker Avance 300, Bruker Avance III HD 400, or a Bruker Avance II^+^ 600 spectrometer. Chemical shifts (δ) are reported in parts per million (ppm) referenced to tetramethylsilane (0.00 ppm) as an internal standard for samples in CDCl_3_, or to the respective solvent for samples in DMSO-d_6_ (2.50 ppm). ^13^C NMR spectra were referenced to the respective solvent signals (CDCl_3_, 77.16 ppm). High-resolution mass spectra were acquired on a quadrupole orthogonal acceleration time-of-flight mass spectrometer (Synapt G2 HDMS, Waters, Milford, MA, USA). Melting points (not corrected) were determined using a Reichert Thermovar apparatus.

UV–Vis absorption spectra were recorded on a PerkinElmer (Shelton, CT, USA) Lambda 950 spectrophotometer using blank correction. Fluorescence and excitation spectra were recorded on a HORIBA Jobin Yvon Fluorolog FL3-22 fluorimeter (HORIBA, Minami ku, Japan), and corrections for the excitation beam intensity, the wavelength-dependent sensitivity of the detector, and the optical path were applied.

### 3.3. Synthesis

#### 3.3.1. General Procedure for Palladium-Catalyzed Reaction 1

To an oven-dried reaction tube equipped with a magnetic stirring bar **HetATAP 1** (0.2 mmol, 1 equivalent), boronic acids (0.24 mmol, 1.2 equivalent), Pd(OAc)_2_ (27 mg, 0.12 mmol, 0.6 equivalent), CuI (15.2 mg, 0.08 mmol, 0.4 equivalent), SPhos (10 mg, 0.023, 0.12 equivalent) and DMF (2 mL) were added. The mixture was left and stirred for 12 h at 60 °C in an aluminum heating block. The crude reaction mixture was dissolved in ethyl acetate (20 mL) and washed with water (2 × 20 mL) and brine (1 × 20 mL). The organic layer was subsequently dried over magnesium sulfate and concentrated under reduced pressure. Further purification by column chromatography, using a PE-DCM gradient as the eluent, afforded the solid compounds. 

#### 3.3.2. Palladium-Catalyzed Reaction 2 of Compound **HetATAP 1**

**HetATAP 1** (24.3 mg, 0.1 mmol, 1 equivalent), PhSnBu_3_ (44.2 mg, 0.12 mmol, 1.2 equivalent), and Pd_2_dba_3_ (5 mg, 0.005, 0.05 equivalent) were added to an oven-dried reaction tube equipped with a magnetic stirring bar and dissolved in toluene (1 mL) under a nitrogen atmosphere. The reaction mixture was left and stirred at 70 °C for 24 h. The reaction mixture was diluted with EtOAc (20 mL) and washed with water (2 × 20 mL) and brine (1 × 20 mL). The organic layer was subsequently dried over magnesium sulfate and concentrated under reduced pressure. Further purification by column chromatography, using a PE-DCM (1:1–0:1) gradient as the eluent, afforded a yellow solid **HetATAP 2** (4 mg, 13%).

#### 3.3.3. General Procedure for Palladium-Catalyzed Reaction 3

To an oven-dried reaction tube equipped with a magnetic stirring bar **HetATAP 1** (0.2 mmol, 1 equivalent), bromoarene reagents (0.2 mmol, 1.0 equivalent), Pd(OAc)_2_ (2.4 mg, 0.01 mmol, 0.05 equivalent), Cy_3_HBF_4_ (7.8 mg, 0.02 mmol, 0.1 equivalent), pivalic acid (6.6 mg, 0.03, 0.3 equivalent), K_2_CO_3_ (88 mg, 0.6 mmol, 3.0 equivalent) and dry toluene (4 mL) were added. The mixture was left and stirred for 24 h at 110 °C in an aluminum heating block. The reaction mixture was dissolved in ethyl acetate (20 mL) and washed with water (2 × 20 mL) and brine (1 × 20 mL). The organic layer was subsequently dried over magnesium sulfate and concentrated under reduced pressure. Further purification by column chromatography, using a PE-DCM gradient as the eluent, afforded the solid compounds.

#### 3.3.4. Experimental Details and Characterization Data

**HetATAP 1**: Yellow solid. Yield 89.5 mg, 74%. Mp 192–195 °C. ^1^H NMR (400 MHz, CDCl_3_) δ 8.35 (d, *J* = 5.5 Hz, 1H), 7.98 (s, 1H), 7.80–7.73 (m, 1H), 7.67 (d, *J* = 8.5 Hz, 1H), 7.48 (d, *J* = 7.0 Hz, 2H), 7.33 (d, *J* = 8.5 Hz, 1H). ^13^C NMR (101 MHz, CDCl_3_) δ 138.77, 138.40, 135.91, 125.03, 124.57, 123.66, 123.55, 121.16, 120.60, 118.70, 111.37, 98.33. HRMS (ESI-Q-TOF): *m*/*z* [M + H]^+^ calculated for C_12_H_7_Cl_1_N_4_: 243.0432, found: 243.0431. 

**HetATAP 2**: (1) Prepared following the general procedure for palladium-catalyzed reaction 1: **HetATAP 1** (0.2 mmol, 1 equivalent), phenylboronic acid (0.24 mmol, 1.2 equivalent), Pd(OAc)_2_ (27 mg, 0.12 mmol, 0.6 equivalent), CuI (15.2 mg, 0.08 mmol, 0.4 equivalent), SPhos (10 mg, 0.023, 0.12 equivalent) and DMF (2 mL), 12 h. Purification by column chromatography, using a PE-DCM gradient (1:1–0:1) as the eluent, afforded **HetATAP 2** (38 mg, 53%) as a yellow solid. (2). Prepared following the procedure for palladium-catalyzed reaction 2: **HetATAP 1** (24.3 mg, 0.1 mmol, 1 equivalent), PhSnBu_3_ (44.2 mg, 0.12 mmol, 1.2 equivalent), Pd_2_dba_3_ (5 mg, 0.005, 0.05 equivalent), 24 h. Purification by column chromatography, using a PE-DCM gradient (1:1–0:1) as the eluent afforded **HetATAP 2** (4 mg, 13%). (3). Prepared following the general procedure for palladium-catalyzed reaction 3: **HetATAP 1** (0.2 mmol, 1 equivalent), bromobenzene (0.2 mmol, 1.0 equivalent), Pd(OAc)_2_ (2.4 mg, 0.01 mmol, 0.05 equivalent), Cy_3_HBF_4_ (7.8 mg, 0.02 mmol, 0.1 equivalent), pivalic acid (6.6 mg, 0.03, 0.3 equivalent), K_2_CO_3_ (88 mg, 0.6 mmol, 3.0 equivalent) and dry toluene (4 mL), 24 h. Purification by column chromatography, using a PE-DCM (1:1–0:1) gradient as the eluent, afforded the solid compounds **HetATAP 2** (42 mg, 66%) as a yellow solid. Mp 192–194 °C. ^1^H NMR (400 MHz, CDCl_3_) δ 8.52–8.40 (m, 1H), 8.35 (dd, *J* = 8.5, 1.2 Hz, 2H), 8.22–8.09 (m, 1H), 7.75 (d, *J* = 8.5 Hz, 1H), 7.67–7.59 (m, 2H), 7.61–7.52 (m, 2H), 7.48–7.38 (m, 1H), 7.35 (d, *J* = 8.5 Hz, 1H). ^13^C NMR (101 MHz, CDCl_3_) δ 138.90, 138.71, 129.12, 128.14, 127.88, 126.57, 125.28, 124.00, 123.90, 123.02, 121.22, 120.83, 119.15, 111.53. HRMS (ESI-Q-TOF): *m*/*z* [M + H]^+^ calculated for C_18_H_11_ClN_4_: 319.0744, found: 319.0741.

**HetATAP 3**: (1). Prepared following the general procedure for palladium-catalyzed reaction 1: **HetATAP 1** (0.2 mmol, 1 equivalent), 4-methoxyphenylboronic acid (0.24 mmol, 1.2 equivalent), Pd(OAc)_2_ (27 mg, 0.12 mmol, 0.6 equivalent), CuI (15.2 mg, 0.08 mmol, 0.4 equivalent), SPhos (10 mg, 0.023, 0.12 equivalent) and DMF (2 mL), 12 h. Purification by column chromatography, using a PE-DCM gradient (1:1–0:1) as the eluent, afforded HetATAP**3** (32.9 mg, 47%) as a yellow solid. (2). Prepared following the general procedure for palladium-catalyzed reaction 3: **HetATAP 1** (0.2 mmol, 1 equivalent), 4-bromoanisole (0.2 mmol, 1.0 equivalent), Pd(OAc)_2_ (2.4 mg, 0.01 mmol, 0.05 equivalent), Cy_3_HBF_4_ (7.8 mg, 0.02 mmol, 0.1 equivalent), pivalic acid (6.6 mg, 0.03, 0.3 equivalent), K_2_CO_3_ (88 mg, 0.6 mmol, 3.0 equivalent) and dry toluene (4 mL), 24 h. Purification by column chromatography, using a PE-DCM (1:1–0:1) gradient as the eluent, afforded the solid compounds **HetATAP 3** (43.4 mg, 62%) as a yellow solid. Mp 248–250 °C. 1H NMR (400 MHz, CDCl_3_) δ 8.45–8.35 (m, 1H), 8.26 (d, *J* = 8.9 Hz, 1H), 8.11–8.06 (m, 1H), 7.69 (d, *J* = 8.5 Hz, 1H), 7.57–7.48 (m, 1H), 7.31 (d, *J* = 8.5 Hz, 1H), 7.15 (d, *J* = 8.9 Hz, 1H). ^13^C NMR (101 MHz, CDCl_3_) δ 159.28, 138.93, 138.37, 136.12, 129.97, 128.19, 125.05, 123.93, 122.88, 121.04, 120.61, 120.33, 119.11, 114.65, 111.49, 110.98, 55.45. HRMS (ESI-Q-TOF): *m*/*z* [M] calculated for C_19_H_13_ClN_4_O: 348.0778, found: 348.0778. 

**HetATAP 4**: (1). Prepared following the general procedure for palladium-catalyzed reaction 1: **HetATAP 1** (0.2 mmol, 1 equivalent), 4-ethoxycarbonylphenylboronic acid (0.24 mmol, 1.2 equivalent), Pd(OAc)_2_ (27 mg, 0.12 mmol, 0.6 equivalent), CuI (15.2 mg, 0.08 mmol, 0.4 equivalent), SPhos (10 mg, 0.023, 0.12 equivalent) and DMF (2 mL), 12 h. Purification by column chromatography, using a PE-DCM gradient (1:1–0:1) as the eluent, afforded **HetATAP 4** (27.3 mg, 35%) as a yellow solid. (2). Prepared following the general procedure for palladium-catalyzed reaction 3: **HetATAP 1** (0.2 mmol, 1 equivalent), ethyl 4-bromobenzoate (0.2 mmol, 1.0 equivalent), Pd(OAc)_2_ (2.4 mg, 0.01 mmol, 0.05 equivalent), Cy_3_HBF_4_ (7.8 mg, 0.02 mmol, 0.1 equivalent), pivalic acid (6.6 mg, 0.03, 0.3 equivalent), K_2_CO_3_ (88 mg, 0.6 mmol, 3.0 equivalent) and dry toluene (4 mL), 24 h. Purification by column chromatography, using a PE-DCM (1:1–0:1) gradient as the eluent, afforded the solid compounds **HetATAP 4** (39.8 mg, 51%) as a yellow solid. Mp 249–251 °C. ^1^H NMR (400 MHz, CDCl_3_) δ 8.53–8.48 (m, 2H), 8.48–8.43 (m, 1H), 8.29–8.24 (m, 3H), 8.24–8.15 (m, 1H), 7.88 (dd, *J* = 8.2, 1.3 Hz, 1H), 7.64–7.48 (m, 2H), 7.39 (dd, *J* = 8.2, 4.8 Hz, 1H), 4.44 (q, *J* = 7.1 Hz, 2H), 1.45 (t, *J* = 7.1 Hz, 3H). ^13^C NMR (101 MHz, CDCl_3_) δ 166.23, 139.56, 139.37, 137.33, 132.86, 130.24, 128.54, 125.55, 125.39, 124.20, 123.92, 123.09, 121.26, 119.58, 119.00, 111.41, 109.35, 61.04, 14.41. HRMS (ESI-Q-TOF): *m*/*z* [M + H]^+^ calculated for C_21_H_15_ClN_4_O_2_: 391.0956, found: 391.0954.

**HetATAP 5**: (1). Prepared following the general procedure for palladium-catalyzed reaction 1: **HetATAP 1** (0.2 mmol, 1 equivalent), 4-(dimethylamino)phenylboronic acid (0.24 mmol, 1.2 equivalent), Pd(OAc)_2_ (27 mg, 0.12 mmol, 0.6 equivalent), CuI (15.2 mg, 0.08 mmol, 0.4 equivalent), SPhos (10 mg, 0.023, 0.12 equivalent) and DMF (2 mL), 12 h. Purification by column chromatography, using a PE-DCM gradient (1:1–0:1) as the eluent, afforded **HetATAP 5** (22.1 mg, 30%) as a yellow solid. (2). Prepared following the general procedure for palladium-catalyzed reaction 3: **HetATAP 1** (0.2 mmol, 1 equivalent), 1-bromo-4-(dimethylamino)benzene (0.2 mmol, 1.0 equivalent), Pd(OAc)_2_ (2.4 mg, 0.01 mmol, 0.05 equivalent), Cy_3_HBF_4_ (7.8 mg, 0.02 mmol, 0.1 equivalent), pivalic acid (6.6 mg, 0.03, 0.3 equivalent), K_2_CO_3_ (88 mg, 0.6 mmol, 3.0 equivalent) and dry toluene (4 mL), 24 h. Purification by column chromatography, using a PE-DCM (1:1–0:1) gradient as the eluent, afforded the solid compounds **HetATAP 5** (53.8 mg, 73%) as an orange–yellow solid. Mp 115–117 °C. ^1^H NMR (300 MHz, CDCl_3_) δ 8.49–8.26 (m, 1H), 8.19 (d, *J* = 8.5 Hz, 2H), 8.17–7.98 (m, 1H), 7.64 (d, *J* = 8.5 Hz, 1H), 7.58–7.45 (m, 2H), 7.28 (d, *J* = 8.0 Hz, 1H), 6.93 (d, *J* = 8.5 Hz, 2H), 3.06 (s, 6H). ^13^C NMR (101 MHz, CDCl_3_) δ 149.99, 136.30, 127.88, 124.75, 124.01, 123.88, 122.85, 120.84, 119.79, 119.51, 115.65, 112.52, 111.44, 40.41. HRMS (ESI-Q-TOF): *m*/*z* [M] calculated for C_20_H_16_ClN_5_: 361.1094, found: 361.1090. 

## 4. Conclusions

In summary, novel heteroaryl-fused triazapentalene derivatives were successfully synthesized using palladium-catalyzed reactions. The yields of palladium-catalyzed reaction 1 were suboptimal, whereas the direct CH arylation reaction proved to be an effective strategy. The photophysical properties of **HetATAPs 2**–**5** revealed fluorescence in both the solid-state and DW99 solution. However, in organic solvents, only **HetATAP 3** exhibited weak fluorescence in THF. Notably, incorporating a C-4-phenylethoxycarbonyl group on the indazole moiety of **HetATAP** significantly enhanced electron delocalization in the resonance structure, resulting in relatively high fluorescent quantum yields in both the solid-state and DW99 solution. Furthermore, an investigation of the aggregation-induced emission behavior of **HetATAPs 2**–**5** demonstrated that all these materials exhibited AIE properties, highlighting the potential of this approach for developing new organic solid fluorescent materials. 

## Figures and Tables

**Table 1:** The reaction yields of various pathways towards **HetATAPs 2–5**.

HetATAP	R	Yield (%)		
		a	b	c
**2**	H	53	13	66
**3**	OMe	47	- ^a^	62
**4**	COOEt	35	- ^a^	51
**5**	N(Me)_2_	30	- ^a^	73

^a^ Reaction was not carried out. Experimental conditions: (**a**) palladium-catalyzed reaction 1: **HetATAP 1** (0.2 mmol, 1 equivalent), boronic acids (0.24 mmol, 1.2 equivalent), Pd(OAc)_2_ (27 mg, 0.12 mmol, 0.6 equivalent), CuI (15.2 mg, 0.08 mmol, 0.4 equivalent), SPhos (10 mg, 0.023, 0.12 equivalent), and DMF (2 mL), 12 h; (**b**) palladium-catalyzed reaction 2: **HetATAP 1** (24.3 mg, 0.1 mmol, 1 equivalent), PhSnBu_3_ (44.2 mg, 0.12 mmol, 1.2 equivalent), Pd_2_dba_3_ (5 mg, 0.005, 0.05 equivalent), 24 h; (**c**) palladium-catalyzed reaction 3: **HetATAP 1** (0.2 mmol, 1 equivalent), bromoarene reagents (0.2 mmol, 1.0 equivalent), Pd(OAc)_2_ (2.4 mg, 0.01 mmol, 0.05 equivalent), Cy_3_HBF_4_ (7.8 mg, 0.02 mmol, 0.1 equivalent), pivalic acid (6.6 mg, 0.03, 0.3 equivalent), K_2_CO_3_ (88 mg, 0.6 mmol, 3.0 equivalent), and dry toluene (4 mL), 24 h.

**Table 2:** Fluorescence properties of **HetATAPs 1**–**5** in the solid-state.

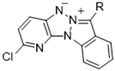	λ_abs,max_ (nm)	λ_em,max_ (nm)	Stokes Shift (cm^−1^)	Φ_F_ (%)
**HetATAP1**	460	584	4600 ± 50	10
**HetATAP2**	525	576	1680 ± 50	5
**HetATAP3**	520	570	1680 ± 50	12
**HetATAP4**	520	571	1710 ± 50	20
**HetATAP5**	485	588	3610 ± 50	5

**Table 3:** Photophysical properties of **HetATAPs 1–5** measured in different solutions.

	λ_abs_(nm)/ε (cm^−1^·M^−1^·10^−3^)				λ_ems_ (nm)/Ф_F_ (%)		
	Tol	THF	ACN	DW99	THF	ACN	DW99
**HetATAP** **1**	407/49.4	402/41.1	398/35.4	388/31.7	463/0.2	450/0.2	461/0.5
**HetATAP** **2**	441/35.1	438/38.4	433/36.0	429/10.6	-	-	541/8.0
**HetATAP** **3**	445/30.4	441/34.0	436/30.0	415/14.6	469/0.1	-	541/2.3
**HetATAP** **4**	450/59.3	446/59.3	443/52.9	428/18.0	-	-	568/13.1
**HetATAP** **5**	456/34.9	454/34.1	449/30.7	423/13.1	-	-	565/2.6

## Data Availability

The original contributions presented in the study are included in the article/Supplementary Materials; further inquiries can be directed to the corresponding authors.

## Supplementary Materials

The following supporting information can be downloaded at https://www.mdpi.com/article/10.3390/molecules30010156/s1.
