# Supplementary material for: Heteroaryl-Fused Triazapentalenes: Synthesis and Aggregation-Induced Emission
Source: Molecules. 2025 Jan 3;30(1):156. doi: 10.3390/molecules30010156 (PMC11721409; doi:10.3390/molecules30010156)
Supplement: Supplementary file 1 [file molecules-30-00156-s001.zip › molecules-3262077-supplementary.pdf]

Heteroaryl-Fused Triazapentalenes: Synthesis and Aggregation-Induced Emission

Yingchun Wang [1,2], Thanh Chung Pham [2], Jianjun Huang [2], Junfeng Wu [1,*] and Wim Dehaen [2,*]

[1] Henan Key Laboratory of Water Pollution Control and Rehabilitation Technology, Henan University of Urban Construction, Pingdingshan 467036, China; wangyc0628@163.com

[2] Sustainable Chemistry for Metals and Molecules, Department of Chemistry, KU Leuven, Celestijnenlaan 200F, 3001 Leuven, Belgium; jianjun.huang@kuleuven.be (J.H.)

* Correspondence: jf8047@163.com (J.W.); wim.dehaen@kuleuven.be (W.D.)

Contents

Synthesis of **HetATAP 1** 2
NMR spectra 3

Synthesis of HetATAP 1

To an oven-dried reaction tube equipped with a magnetic stirring bar were added 2,6-dichloro-3-nitropyridine (192.98 mg, 1 mmol, 1 equiv), indazole (130 mg, 1.1 mmol, 1.1 equiv), cesium carbonate (358.4 mg, 1.1 mmol, 1.1 equiv) and acetonitrile (6 mL). The mixture was left and stirred for 14 hours at 85 °C in an aluminum heating block. The crude reaction mixture was filtered and concentrated, after which it was dissolved in ethyl acetate (15 mL) and washed with water (2 × 20 mL) and brine (1 × 20 mL). The organic layer was subsequently dried over magnesium sulfate and concentrated under reduced pressure. Further purification by column chromatography, using a PE-DCM gradient as the eluent, afforded the 1-(6-chloro-3-nitropyridin-2-yl)-1*H*-indazole 1 as solid. The 1-(6-chloro-3-nitropyridin-2-yl)-1*H*-indazole **1** (0.5 mmol, 1 equiv) were added to an oven-dried reaction tube equipped with a magnetic stirring bar and dissolved in $P(OEt)_3$ (2.5 mL). The microwave assisted reaction was kept at 140 °C for 105 minutes. After removing the solvent under reduced pressure, the mixture was diluted with EtOAc (1 × 20 mL), and washed with water (2 × 20 mL) and brine (1 × 20 mL). The organic layer was subsequently dried over magnesium sulfate and concentrated under reduced pressure. Further purification by column chromatography, using DCM as the eluent, afforded the pure **HetATAP 1** (Scheme S1).

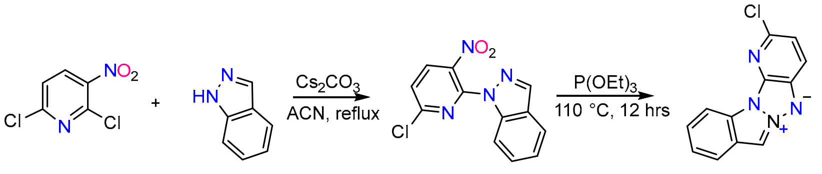

1 **HetATAP 1**

Scheme S1. The synthesis of **HetATAP 1**.

1-(6-chloro-3-nitropyridin-2-yl)-1*H*-indazole **1**: White solid. Yield 146 mg, 53 %. Mp: 168 – 170°C. ^1H NMR (300 MHz, $CDCl_3$) δ 8.81 (d, J = 8.6 Hz, 1H), 8.46 (d, J = 8.9 Hz, 1H), 8.28 (s, 1H), 8.11 (d, J = 8.9 Hz, 1H), 7.80 (d, J = 8.0 Hz, 1H), 7.63 (t, J = 8.1 Hz, 1H), 7.39 (t, J = 7.5 Hz, 1H). HRMS (ESI-Q-TOF): m/z $[M + H]^+$ calcd for $C_{12}H_7Cl_1N_4O_2$: 275.0330, found: 275.0332.

NMR spectra

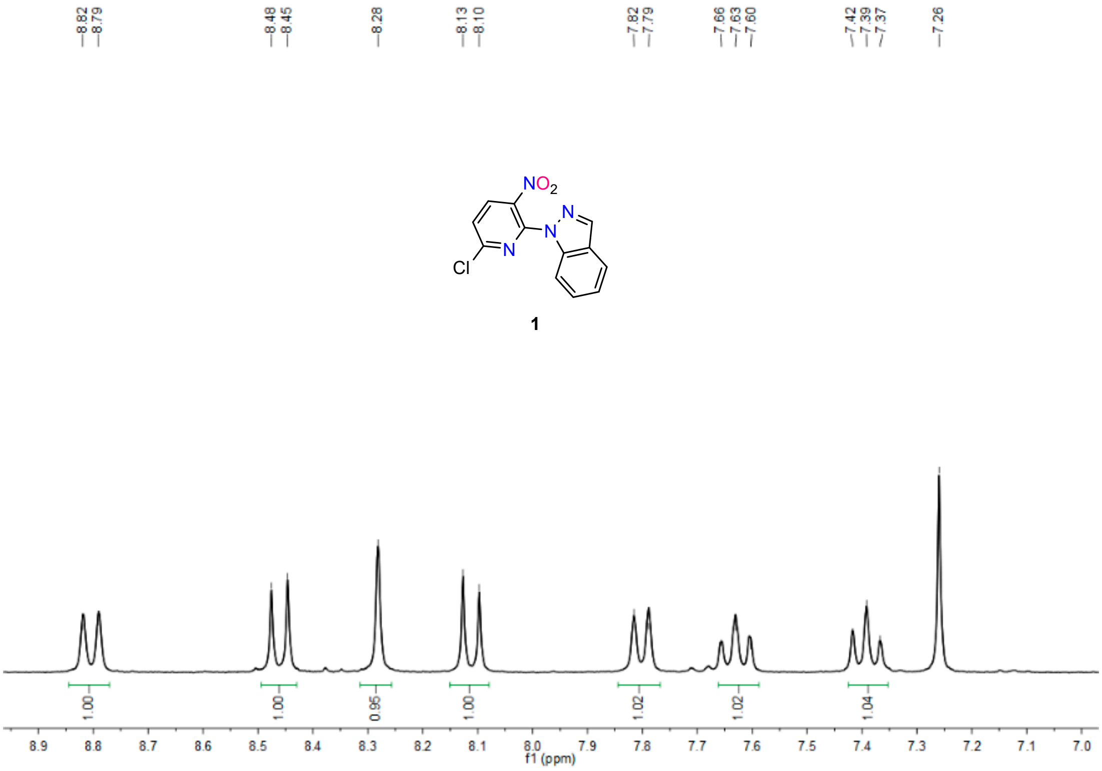
8.82
8.79
8.48
8.45
8.28
8.13
8.10
7.82
7.79
7.66
7.63
7.60
7.42
7.39
7.37
7.26
NO_2
N
N
N
Cl
1
1.00
1.00
0.95
1.00
1.02
1.02
1.04
8.9 8.8 8.7 8.6 8.5 8.4 8.3 8.2 8.1 8.0 7.9 7.8 7.7 7.6 7.5 7.4 7.3 7.2 7.1 7.0
f1 (ppm)

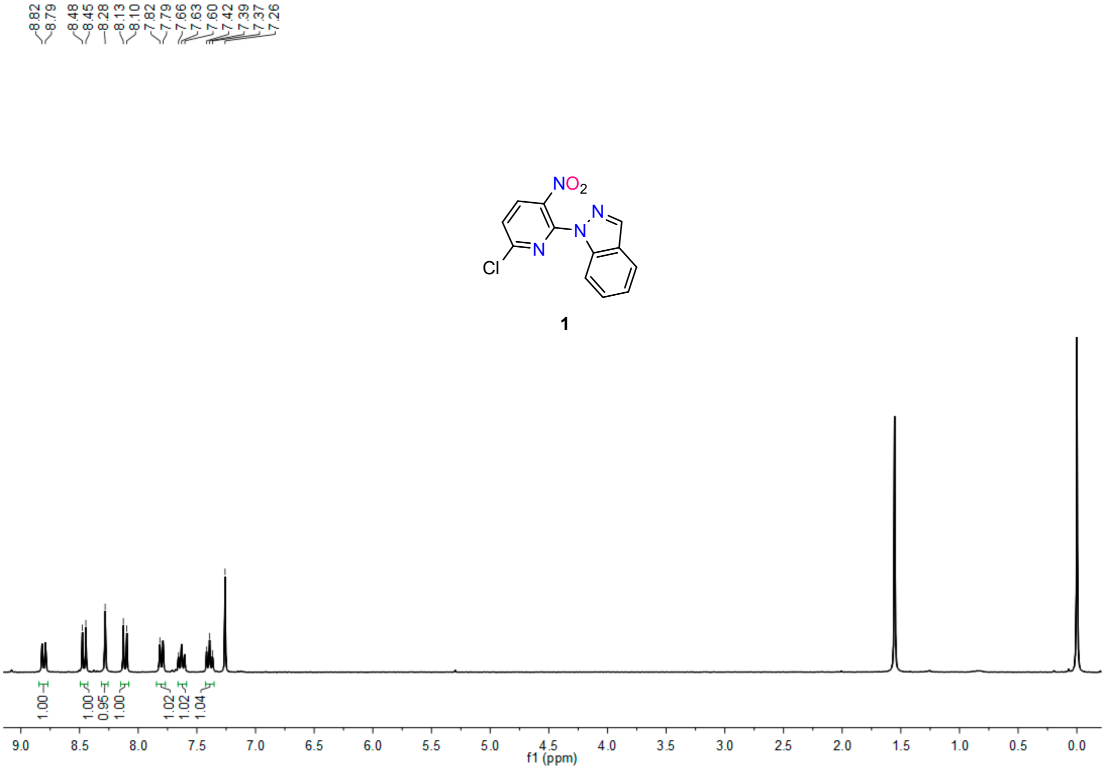

Figure S1. ^{1}H NMR spectrum of (2-nitropyridinyl)-1H-indazole **1** recorded in $CDCl_3$.

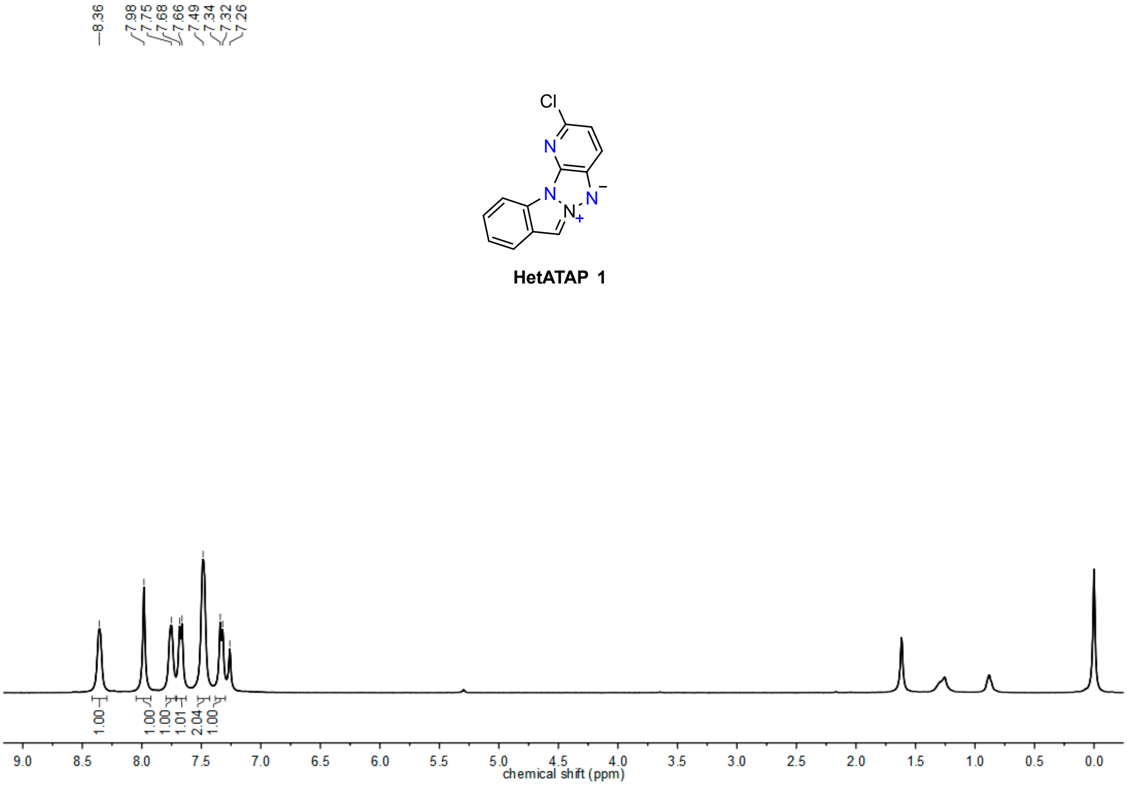

Figure S2. ^{1}H NMR spectrum of **HetATAP 1** recorded in $CDCl_3$.

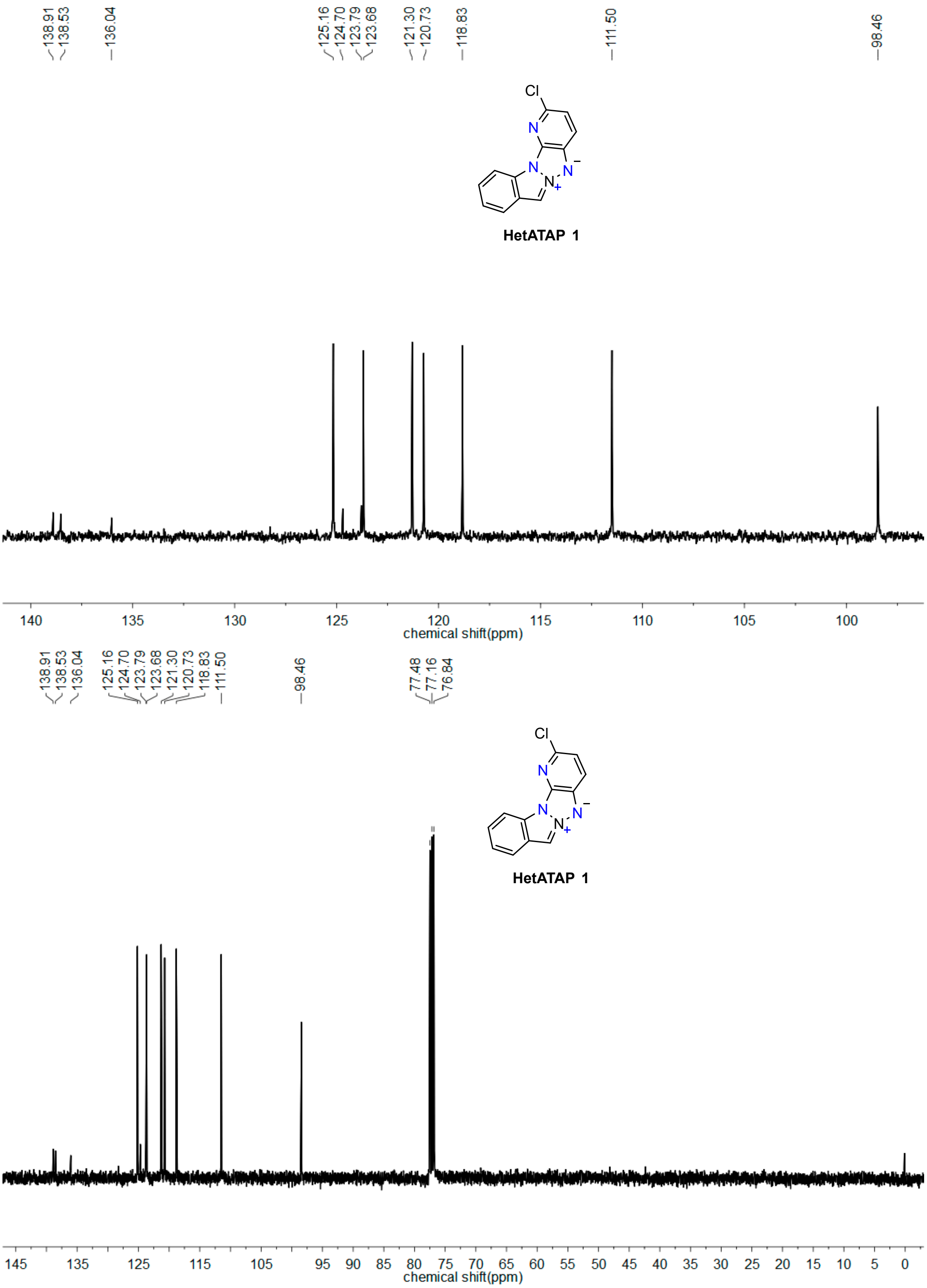

Figure S3. ^{13}C NMR spectrum of **HetATAP 1** recorded in $CDCl_3$.

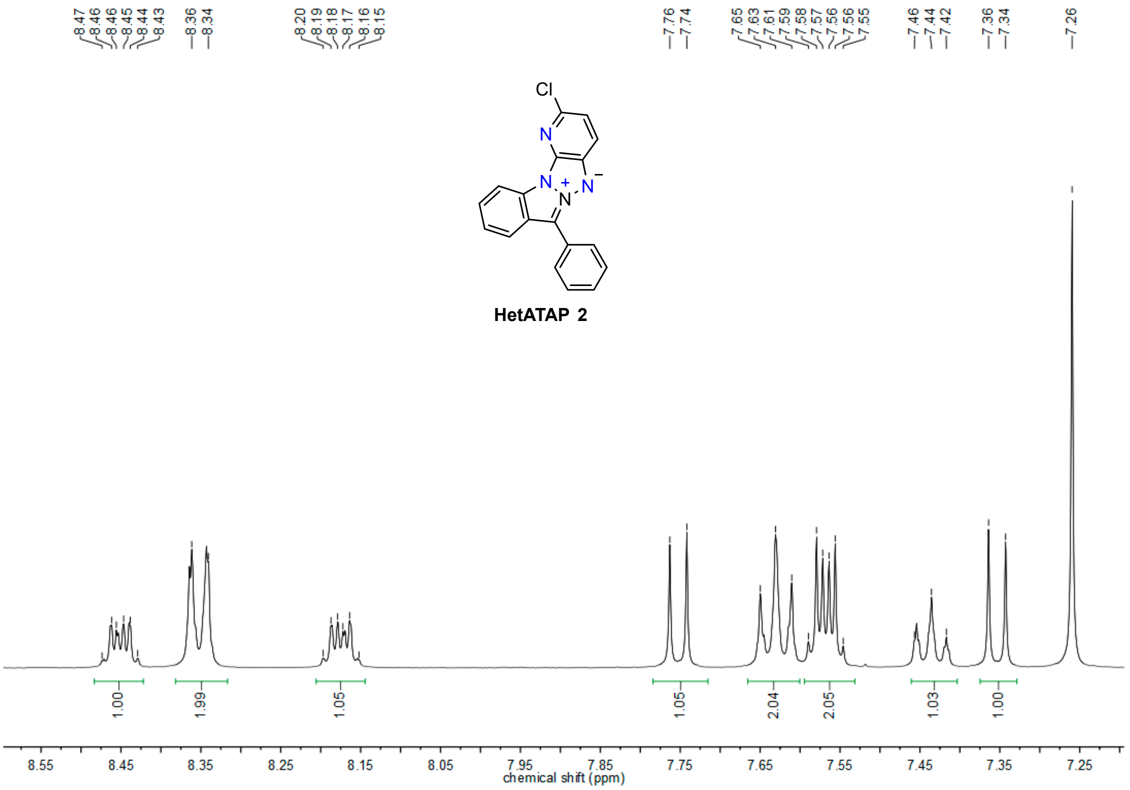

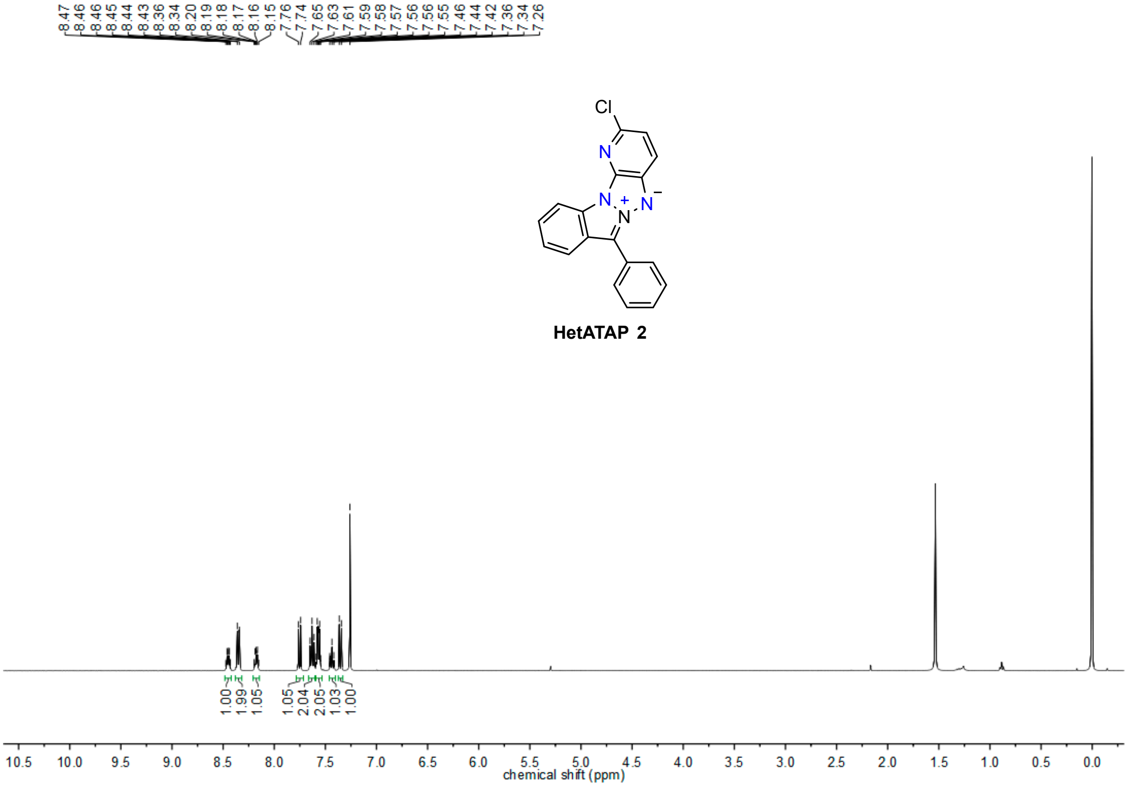

Figure S4. ^{1}H NMR spectrum of **HetATAP 2** recorded in $CDCl_3$.

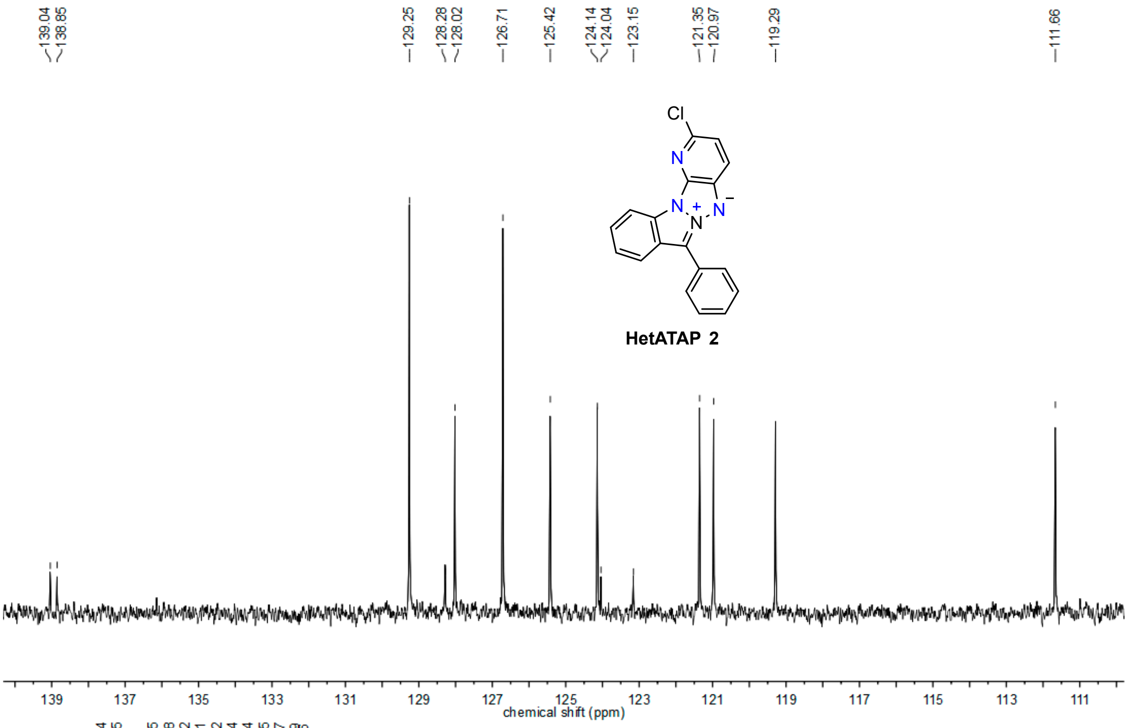

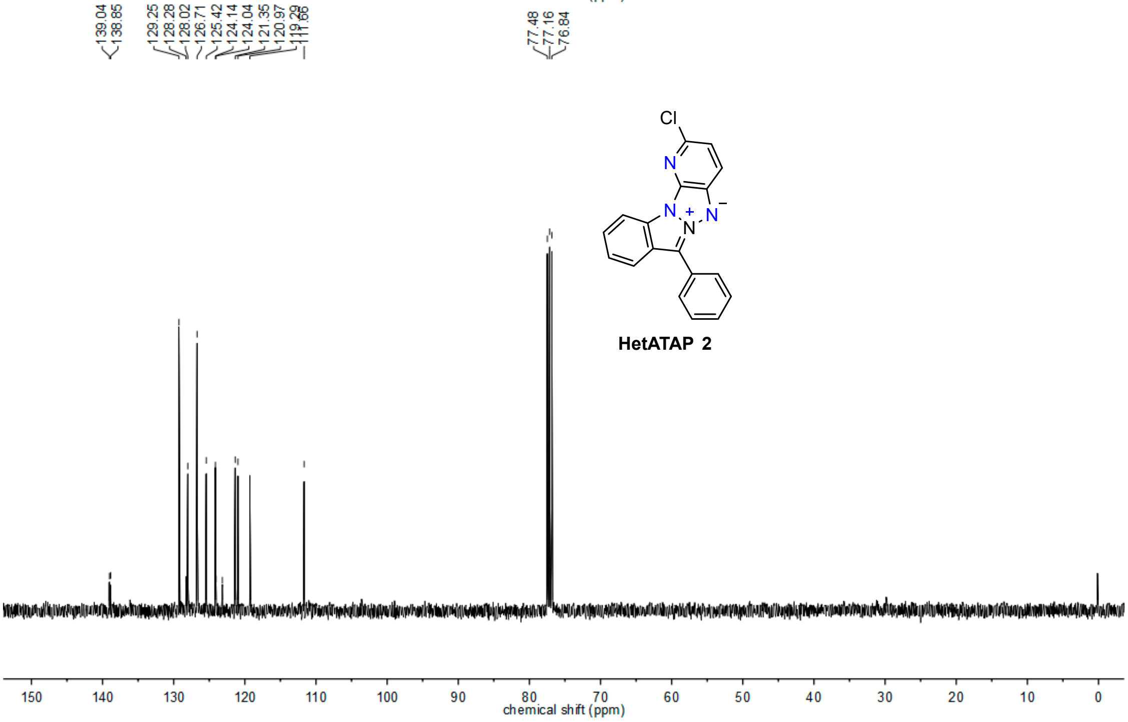

Figure S5. ^{13}C NMR spectrum of **HetATAP 2** recorded in $CDCl_3$.

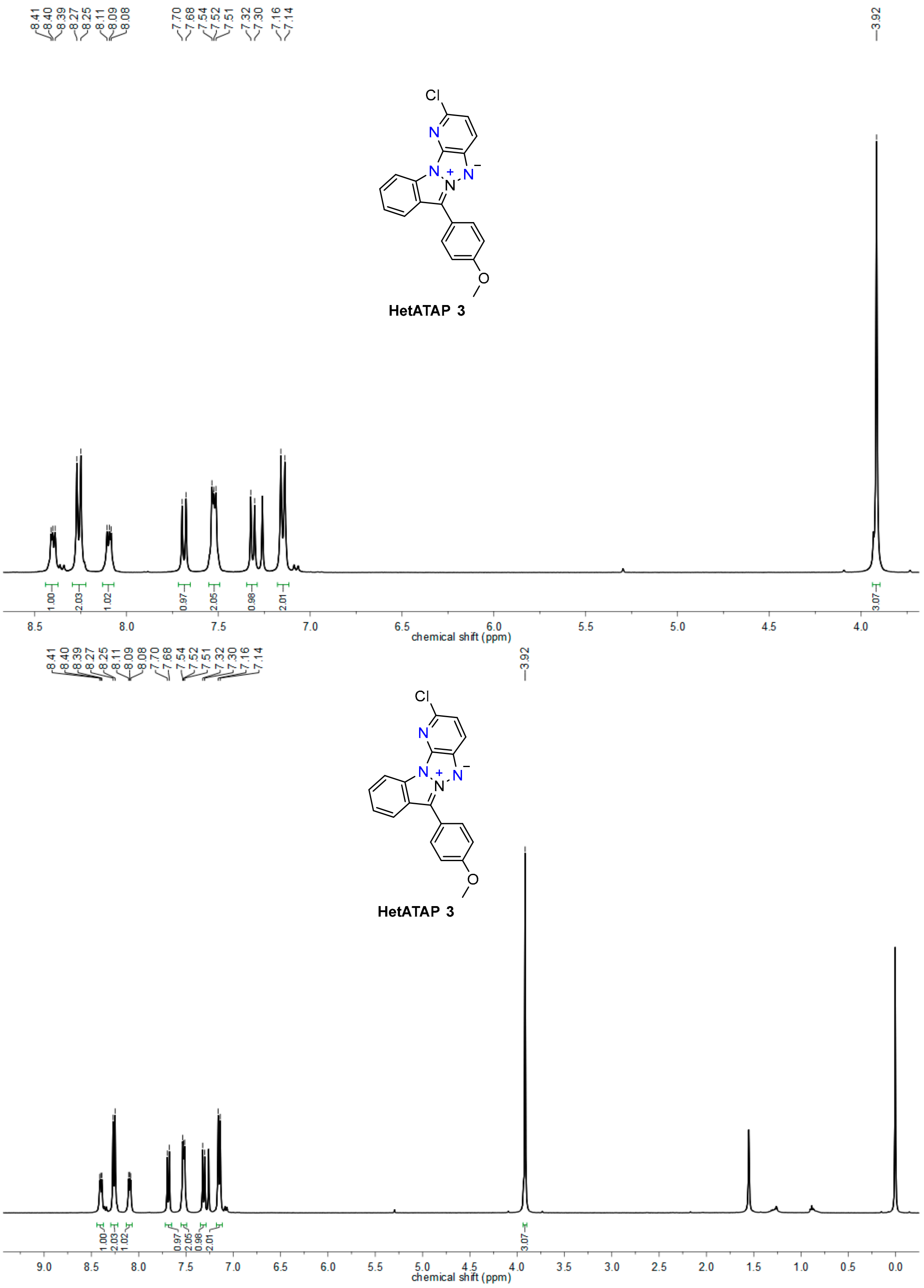

Figure S6. ^{1}H NMR spectrum of **HetATAP 3** recorded in $CDCl_3$.

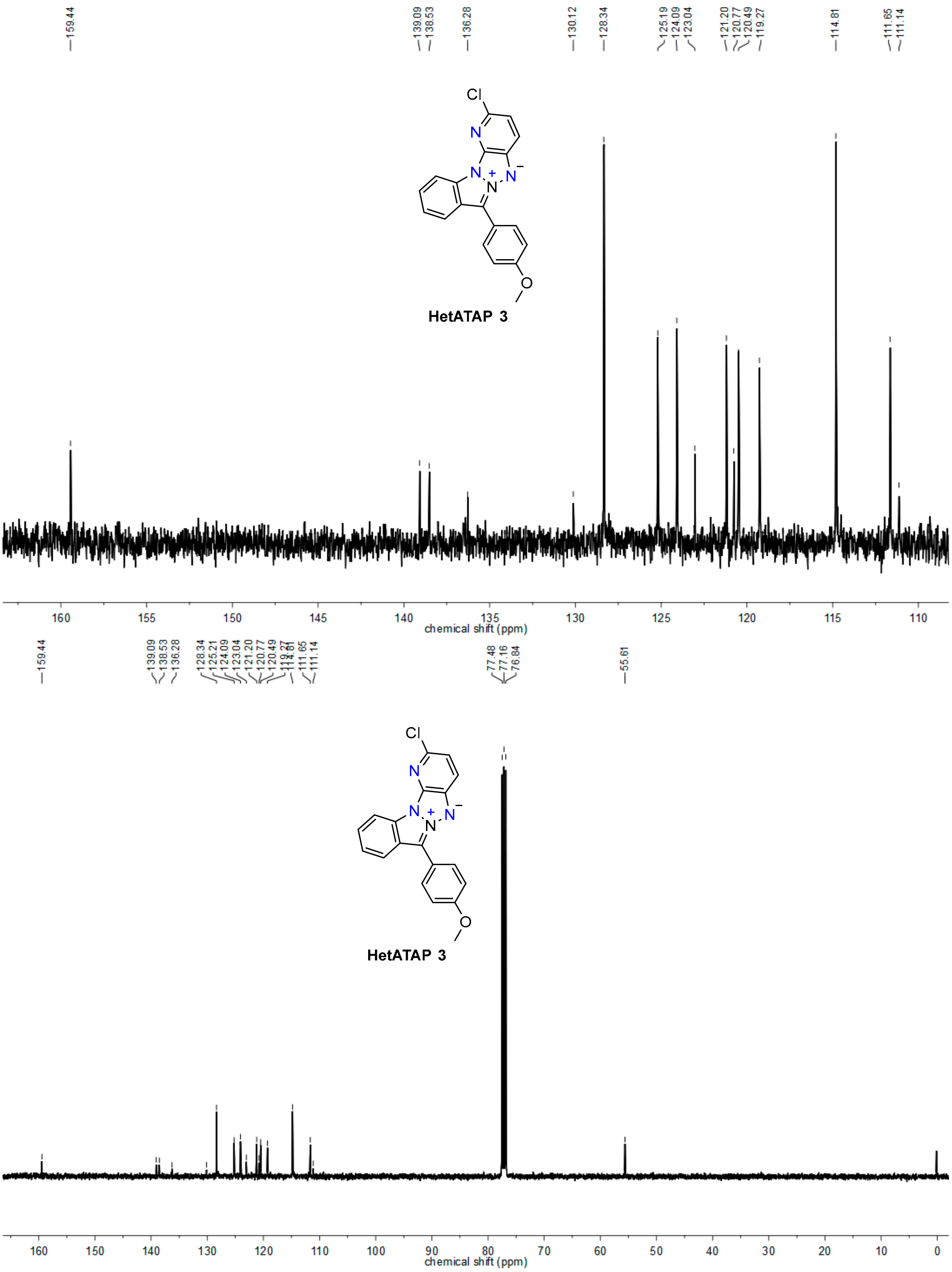

Figure S7. ^{13}C NMR spectrum of **HetATAP 3** recorded in $CDCl_3$.

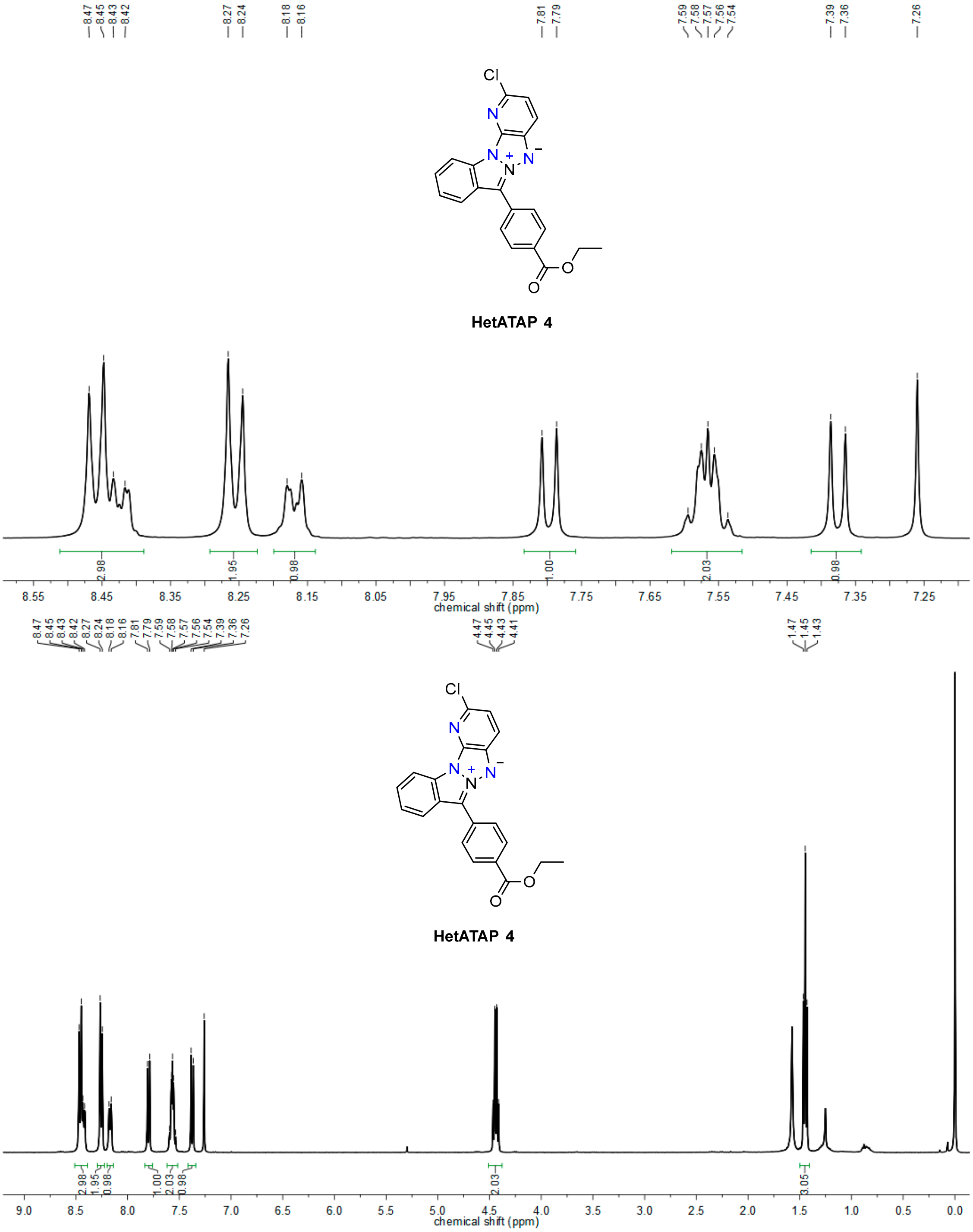

Figure S8. ^{1}H NMR spectrum of **HetATAP 4** recorded in $CDCl_3$.

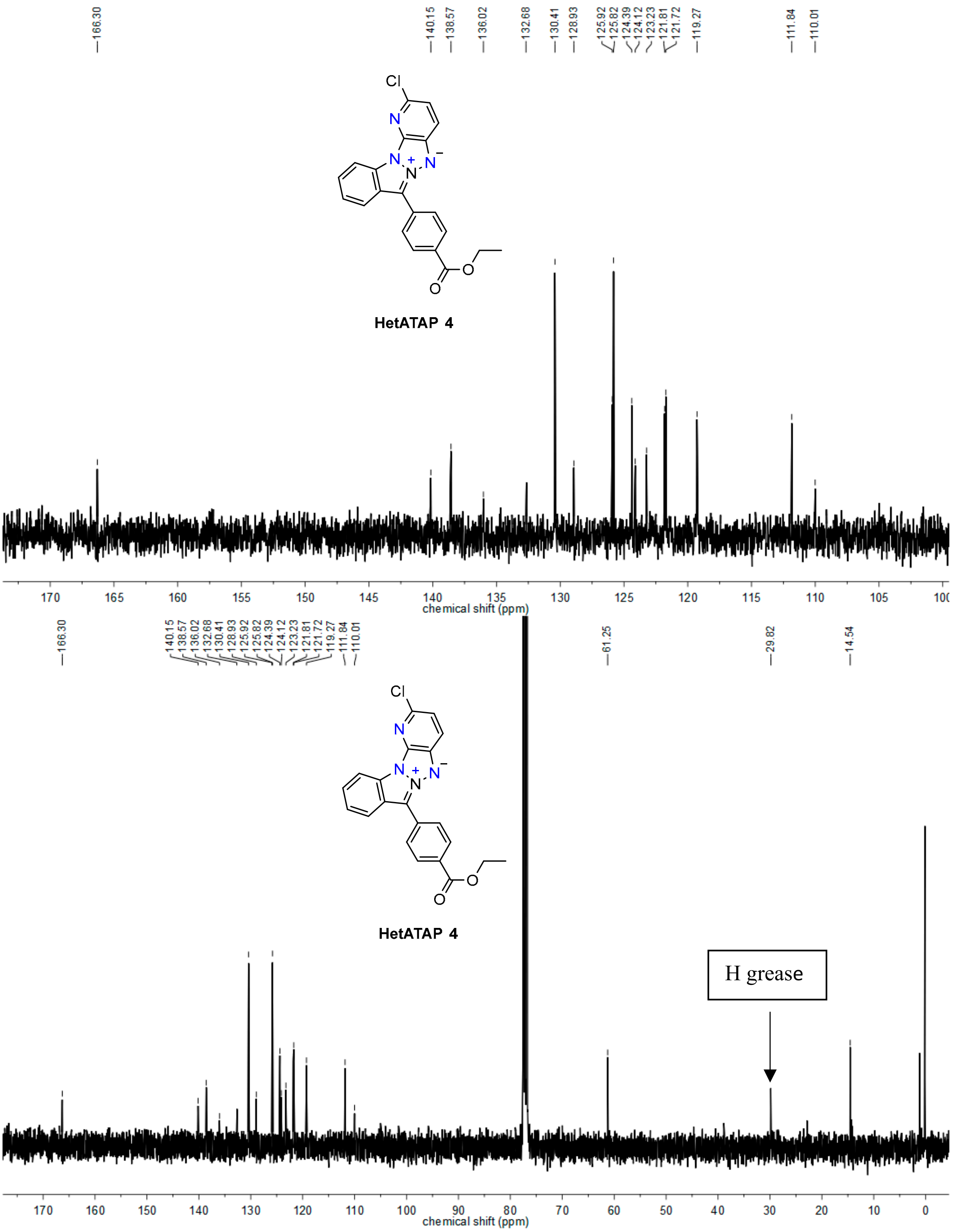

Figure S9. ^{13}C NMR spectrum of **HetATAP 4** recorded in $CDCl_3$.

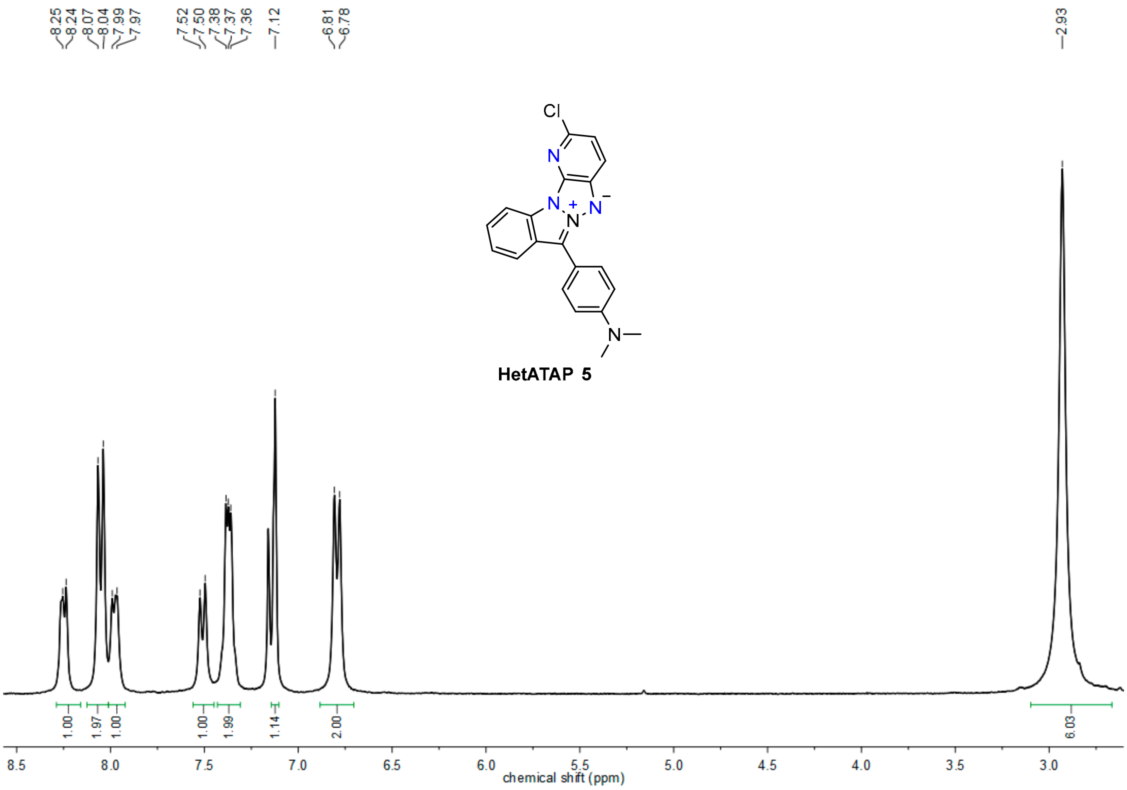

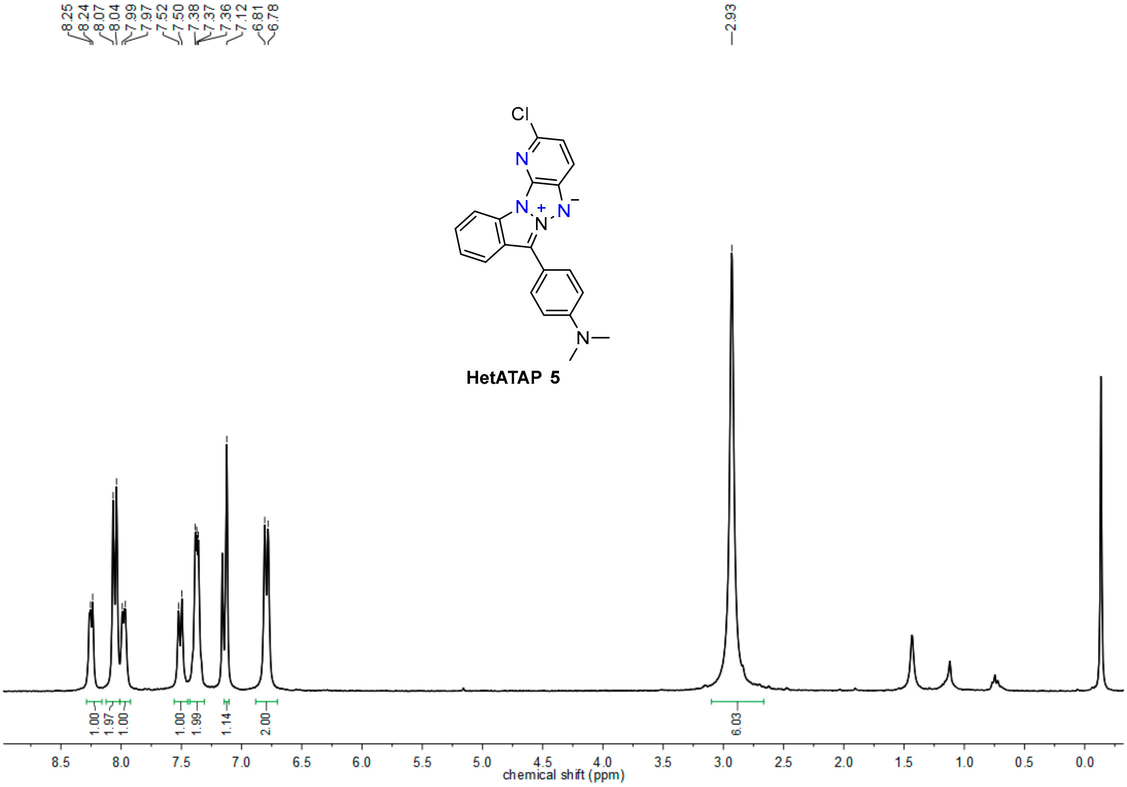

Figure S10. ^{1}H NMR spectrum of **HetATAP 5** recorded in $CDCl_3$.

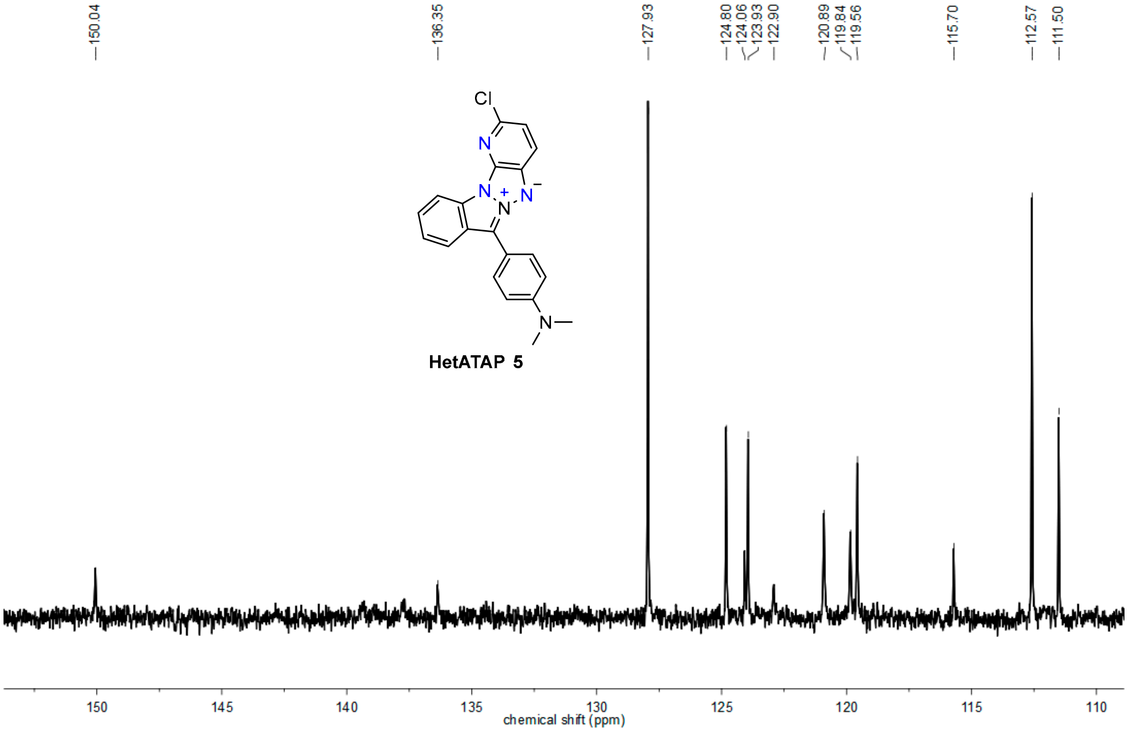
150.04
136.35
127.93
124.80
124.06
123.93
122.90
120.89
119.84
119.56
115.70
112.57
111.50
Cl
N
N
N
N
HetATAP 5
150
145
140
135
130
125
120
115
110
chemical shift (ppm)

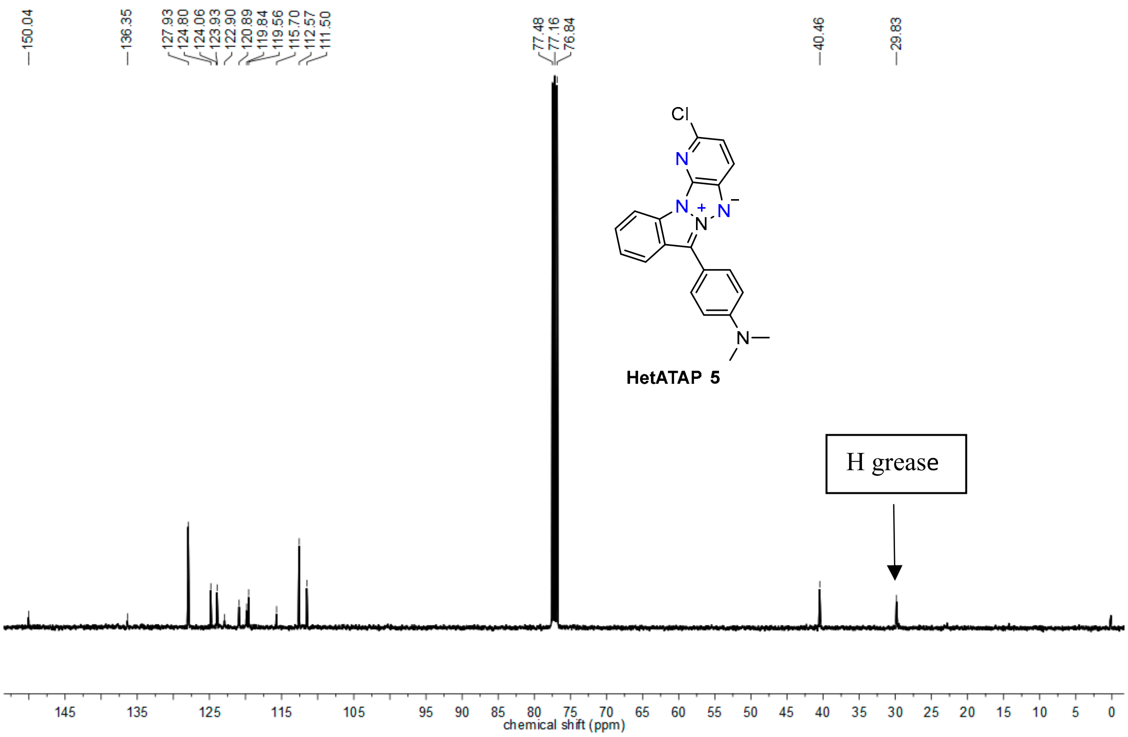

Figure S11. ^{13}C NMR spectrum of **HetATAP 5** recorded in $CDCl_3$.
